# Doctor–patient interactions that exclude patients experiencing homelessness from health services: an ethnographic exploration

**DOI:** 10.3399/BJGPO.2021.0031

**Published:** 2021-05-12

**Authors:** Austin O'Carroll, David Wainwright

**Affiliations:** 1 Programme Director, North Dublin City General Practice Training Programme, Catherine McAuley Centre, Dublin, Republic of Ireland; 2 Doctoral Graduate, University of Bath, Department for Health, Bath, UK; 3 GP, Grangegorman Primary Care Centre, Dublin, Republic of Ireland; 4 Senior Lecturer, University of Bath, Department for Health, Bath, UK

**Keywords:** homeless persons, accessibility of health service, anthropology, cultural, health services

## Abstract

**Background:**

People experiencing homelessness have poor health indices and poor access to health care. Their health service utilisation (HSU) is typified by: late illness presentations; poor attendance rates at appointments; low usage of primary care services and outpatient departments; and high utilisation of emergency departments and inpatient services. Why people experiencing homelessness have these particular HSU patterns is poorly understood.

**Aim:**

This research sought to explore barriers to health service usage for people experiencing homelessness.

**Design & setting:**

The authors conducted critical realist ethnography over 13 months in Dublin with people experiencing homelessness at four purposively chosen sites (a food hall, a drop-in centre, an emergency department, and an outreach service for rough sleepers).

**Method:**

Ethnographic research was supplemented with focus groups of hospital doctors and people experiencing homelessness, and with 50 semi-structured interviews with people experiencing homelessness. The epistemological framework was critical realism.

**Results:**

One of the factors identified in the research as contributing to the HSU pattern of people experiencing homelessness was recurrent interactions between health professionals and patients, whereby patients were either excluded or discouraged from attending health services, or self-excluded themselves from services. These interactions were described as ’conversations of exclusion’. Four such conversations were described: ‘the benzodiazepine conversation‘; ‘the mistrustful conversation‘; ‘the blaming conversation‘; and ‘the assertive conversation’.

**Conclusion:**

There are certain recurrent interactions between people experiencing homelessness and doctors that result in the exclusion of people experiencing homelessness from health services.

## How this fits in

Despite their significant morbidity burden, people experiencing homelessness face difficulties accessing health care. This article identifies a new communicative barrier called ‘conversations of exclusion‘. The identification of this barrier raises the possibility of developing communication skills that would remove the barrier. An example of a communication skill to transform the benzodiazepine conversation of exclusion to one of inclusion is described.

## Introduction

People experiencing homelessness have poor health and mortality indices.^1–13^ Despite this, they make poor use of health services.^14–16^ For example, they tend to: present late in the course of illness;^14,17–19^ default from services before treatment is completed or miss appointments;^14,16,18,20–22^ and be high utilisers of secondary care services and low utilisers of general practice.^14,17,23^ The literature outlines numerous reasons why people experiencing homelessness have this particular HSU pattern. These include primary and secondary care barriers they face.^8,12,14,24–29^ Such barriers include physical barriers (for example, distance to travel to their GP); administrative barriers (for example, filling in application forms or appointments, which are either not received or not attended owing to the chaotic nature of homelessness); communicative barriers (for example, non-health literacy proofed materials); attitudinal barriers (owing to homelessness and substance-misuse stigma); and internalised barriers, which are internal thoughts and/or feelings influenced by the sociocultural environment that result in reduced health service attendance (for example, fatalistic cognitions whereby people experiencing homelessness presume they will die young so question why they should care for their health, or feelings of embarrassment related to their hygiene when sitting in GP practices).^14^

In terms of context, this research focuses on conversations of exclusion, which is a theme identified by the authors in earlier published research.^14^ This article explores how these conversations act as communicative barriers, resulting in patient exclusion from health services.

## Method

The exploration of HSU for people experiencing homelessness has included a variety of approaches including cross-sectional surveys^8,18,30,31^ and grounded theory.^32,33^ To the authors’ knowledge, to date, ethnography has not been used.

This ethnography involved 160 hours of field work in Dublin at four purposively chosen sites between December 2011 and January 2013. These provided a wide typology of people experiencing homelessness (based on: age; homelessness category, for example, hostel-based versus rough sleeping; and health issues, for example, mental health, drug misuse, alcohol misuse, and so on). There were three fixed sites (a drop-in centre, a food hall with an on-site GP service, and an emergency department), while the fourth was with an outreach team working with rough sleepers. The field work was supplemented with 50 semi-structured interviews (see Supplementary Box S1 for interview guide) with participants experiencing homelessness, and two focus groups with substance misusers experiencing homelessness and hospital-based GP trainees. Ethnography can incorporate quantitative and qualitative methods to supplement data-gathering.^34^ In total there were 46 ethnographic participants, 50 semi-structured interviewees, and 26 focus group participants (see Supplementary Table S1 for participant descriptions). Critical realism was the epistemological framework for two reasons. First, homelessness is a constructed notion, yet there is a harsh reality to being homeless that is dangerous to health. Second, the dilemma in choosing a relativist versus realist epistemological approach lies in the fact that the decision to consult lies in the subjective realm, yet there are identifiable consulting patterns for people experiencing homelessness that exist in reality. Critical realism offers an approach whereby the subjective experiencing of illness can be explored while recognising that the actuality of HSU for people experiencing homelessness derives from generative mechanisms.^35^

Data from the field work, semi-structured interviews, and focus groups were recorded and transcribed. Data gathering was terminated once saturation was achieved. The data were coded using NVivo (version 10), using Saldaña’s approach to coding of moving from initial codes to conceptual categories^36^ (see Supplementary Box S2 for coding scheme). For each theme identified, the number of sources referring to that particular theme, as well as number of actual references, were numerated. Credibility is improved by increasing numbers of sources while dependability is improved by increasing numbers of references.^37^

## Results

A series of repeatedly occurring interactions between health professionals and people experiencing homelessness were identified. These interactions tended to result in both parties developing or reinforcing negative opinions and/or stereotypes, and often resulted in the patient being barred or self-excluding themselves from health services. These interactions were termed ‘conversations of exclusion’.

There were four conversations of exclusion identified:

- the benzodiazepine conversation;
- the mistrustful conversation;
- the blaming conversation; and
- the assertive conversation.

### The benzodiazepine conversation (22 sources and 79 references)

This interaction could equally have been called the codeine, morphine, or pregabalin conversation. The benzodiazepine conversation was described by Participant-1, a young girl addicted to heroin and benzodiazepines, who had contracted both HIV and hepatitis C. They described how once they asked for benzodiazepines the doctor would angrily respond. The participant explained how this reaction would result in them becoming annoyed and asking the doctor why not. The conversation would then degenerate into an argument that would end with the participant leaving. Participant-2 said they had gone to doctors and *’once they hear you asking for benzos they get very angry’.*

In this conversation, the person experiencing homelessness who takes benzodiazepines believes doctors should give them to prevent a range of ‘medical conditions’ including anxiety or panic attacks (Participant-3: *’Say if I get panicky, that’s when I take one of me D 5s and I’ll simmer down.’*); withdrawals, including seizures (Partcipant-4: *’Any of them seven seizures I took* [from benzodiazepine withdrawals] *could have killed me*.’); or to help them sleep (Participant-5: *’homelessness is a lot of a strain ... I needed them cos ... it was the only thing that would get me to sleep.’*). For participants, these tablets were extremely important for surviving the rigours of homelessness:

*’Them three would be the world to me. That is all I’d need.’* (Participant-6)

The conversation can also be initiated by the doctor predicting the patient will ask for benzodiazepines. For example:

*’Ah he’d say, “Whatever you do don’t start asking me for Benzos.” It’s just the first thing he’d say and I wouldn’t be asking him for nothing … Like a check up or I can’t breathe … You know from sleeping out in the cold and me hips pained me a lot … I slept rough for four years solid.* [The doctor would say] *“No, I don’t give Benzos.” I say, “I didn’t ask you.” And then you’d get into an argument with him and I’d walk out.’* (Participant-7)

In addition, Participant-8 was asked to leave a doctor when they went for a chest infection for presumed benzo-seeking behaviour *’and I wasn’t even looking for them’*.

The doctor’s presumption that patients will ask for benzodiazapines can also result in the doctor fearing assault, which inhibits the conduction of a proper consultation. For example:

*’The thing at the minute if the doctor hears you’re on drugs — they won’t give anything … Because they think … you’re looking for opiates or Dalmane or Zimovane or whatever it is they take, you know. They think that ... he’s going to hit me at any minute.’* (Participant-4)

That same fear was outlined by one of the doctors in the focus group. For example:

*’They’re not leaving until they get it ... demanding this off a doctor or that off a doctor and the doctor hasn’t got time and they’re getting angry then.’* (Doctor-1)

One participant described how the discrimination would spread owing to doctors sharing stories:

*’Me own doctor ... like ... stopped me benzos and told … me to get another GP. So I found about another doctor and heard he was good … Well I was only in the room when he looked at his notes and said he had to make a phone call … he rang me old doctor… well … at the end he turns to me and says he wasn’t go to give me anything and to leave and never come back ... like.’* (Participant-8)

The benzodiazepine conversation also took place in hospital settings. For example:

*’I went in completely clean and they found I had a drug history. There was a guy that broke his finger and he had a morphine line, they wouldn’t give me so much as a panadol* [that is, paracetamol] *… They thought ... he wants his opiates ... I shattered my heel, my ankle and split the base of my shin with a bit of my ankle bone.’* (Participant-9)

The most powerful barrier was created when the patient internalised this deterrent. They avoided attending the doctor based on the presumption that there was no point. For example:

*’You know like … it’s a fight for me to get up out of bed and get dressed and get out and walk out that door every morning … I have problems in me family, and I’m anxious about that and … I’ve two kids myself. I feel if I went in and told the doctor that, that he’d just turn around and say, “Ah, he’s just looking for fuckin…” you know. So I don’t bother like, you know what I mean … He sees me as a junkie. Just looking out to get more fuckin tablets.’* (Participant-10)

These disputes over benzodiazepines do cause significant difficulties for doctors. However, the conversations result in those most in need of help being excluded. This was captured in one particularly harrowing story:

*’There was a doctor who worked in the inner city who used keep a poodle underneath her desk. A female patient asked for benzodiazepines and was refused* ... [and was barred] ... *so as revenge the patient stole the poodle, shaved it and tied her to the railings outside her surgery. That patient contracted HIV. She became very depressed and attempted suicide by throwing herself in front of a train. The train amputated one of her legs and the other… eventually needed to be amputated. Two years later she threw herself under the train a second time, and died.’* (Participant-11)

### The mistrustful conversation (19 sources and 66 references)

A number of the doctors described feeling disrespected and angry owing to people experiencing homelessness telling lies. For example, one doctor was asked to write a prescription for a patient that they discovered was not legitimate:

*’So I felt very annoyed that someone that I had treated with respect was lying to get the prescription ... You know you do your best, you treat someone with respect and then they turn around and they treat you like that. It will probably make me more suspicious, less trusting.’* (Doctor-2)

Another doctor had been tricked into giving a morphine injection by a man with a fictitious chest pain:

*’So that’s the difficulty. You’re trusting, you have trust there and you do want to treat people. At the same time, your trust has been broken, it can be hard.’* (Doctor-3)

However, for people experiencing homelessness telling a lie was essential to having their needs met. These needs included obtaining drugs:

*‘* *The thing about the drug addiction is ... you do manipulate, you lie and you do coerce when you want that drug. You’ll say mass and you’ll promise the moon.’* (Participant-12)

Other patients who misused drugs lied to avoid facing negative consequences. For example:

*‘* *Well I don’t want to say it to me Methadone doctor ... about drink … Because he’d take me off me takeaways.* *‘* (Participant-13)

Participant-14 told the doctor they were not drinking as they were afraid of losing access to their children. Several patients avoided telling their doctors about drug-related infections (for example, abscesses) or depressive symptoms in case their methadone takeaways were withdrawn or dosage reduced. Three people said how they told lies to be kept in hospital so they could have shelter; for example, one commented:

*’I went in and I told them I’d chest pain, I just wanted to stay the night … They ended up keeping me in for the week.* *‘* (Participant-15)

One participant described whimsically how they would have been better off telling a lie when they had taken an overdose as the doctors would not understand the truth:

*’It probably would have made more sense to say I’d try to kill meself instead of “I just wanted to get high”, you know, it wasn’t a very good answer.* *‘* (Participant-16)

This presumption that trust was essential for a good relationship was not universal. Many key workers reported not taking offence if they were told a lie as they believed it was a necessary survival behaviour. Once the reality that people experiencing homelessness have to tell lies to survive is accepted, then the key worker just needs to non-judgmentally evaluate the information from the client. For example, an experienced key worker said:

*‘The information people give is key to survival, it is how they make money and access services and stay safe and well … they manage the information they give us to shape how we perceive them, where we place them, how we treat them*.*‘* (Participant-17)

### The blaming conversation (nine sources and 57 references)

The blaming interaction is where the health professional blames patients for causing their health problems. For example:

*’They told me in the A&E that they couldn’t take me in because I was a drug addict and I made my own choices.* *’* (Participant-18)

Participant-19 described how they had been rough sleeping with their partner for 4 years and one day they were soaked in a winter shower. The partner became hypothermic and went to hospital. The doctor told the participant as the partner was losing consciousness that: *’It is disgusting the condition he has got himself into.’* Consequently, they swore they would not go back to hospital unless extremely unwell.

Even when the patient’s behaviour was not contributing to their medical problem they were being blamed. For example:

*’I was following up my treatment for me leg. It felt like “what are you still coming in for, you’re a Heroin Addict. You’re still injecting and all that”. I wasn’t injecting into me leg.’* (Participant-20)

One doctor inadvertently owned up to blaming drug users experiencing homelessness for not following up on their medical advice or appointments:

*’I don’t get angry with people but I do get frustrated … Yes, particularly where crossing the line is kind of a frequent thing and they come back again with the same problem ... I’m just like, “Oh why didn’t you get it done? Something terrible could have happened to you.” I suppose it’s that they’re doing themselves a disservice. So you are getting angry.’* (Doctor-5)

The blaming conversation was described by a participant as being rooted in mutual stereotypes and lack of understanding:

*’I think that we generalise too much towards each other as addicts and as doctors. There’s too much — you’ve done this to yourself. If you break it down, every medical issue is self-inflicted. And as regards the drug thing, a lot of addicts get into it ridiculously young, there’s a lack of education and sometimes just plain ignorance because there’s usually an underlying reason for them taking that drug. Usually to avoid or hide bad memories from their lives or childhood or whatnot because usually there are broken homes or socioeconomic things involved.’* (Participant-12)

Another participant described differences in conversation as being related to class:

*’It is a class thing and a lack of education … He made me feel tiny. He spoke down to me, he belittled me and when I tried to let him know that I was taking them to get a high and I didn’t realise they were anti-depressants. Then he started to speak, he used a bit of Latin ... he tried to let me know that, “I’m going to spout a load of Latin at you and let you know you know absolutely jack.“’* (Participant-12)

### The assertive conversation (seven sources and 14 references)

This conversation related to the fact that people from the housed population often have the skills to assert themselves in a polite manner, whereas people experiencing homelessness were prone to asserting themselves in a manner that people who are not homeless would find aggressive. Participant-21 was persuaded to go to the emergency department for assessment of a head injury and one author accompanied them. They walked up to the receptionist in an aggressive manner and immediately got into an argument where the receptionist looked frightened. They got frustrated and started to walk out. If the author had not acted as an intermediary, they would have not received treatment. This interaction demonstrated how outside homelessness, aggression is an ineffective assertiveness approach.

Participant-22 had been taught by their foster home how to be polite: *’I got strung out and all but I always knew like how to speak polite ... and that would help me get things cos people would like me.’* They learnt the language for traversing mainstream health facilities.

### Changing the conversation can promote engagement and protect the relationship (four sources and seven references)

The identification of conversations of exclusion creates the possibility that communication techniques could be developed to convert these into conversations of inclusion. One participant described how doctors often became angry when refusing a request for benzodiazepines and they advised:

*’Letting the patient talk … Not just cutting in and saying, “Well no I can’t give you that, I don’t give that“ ... put it to them at the start in a nice way. “Listen I’m here to help but Benzos is out of the question.“’* (Participant-23)

Other examples of how to change the conversation to promote engagement are as follows. Participant-24 was seeking benzodiazepines, which the doctor declined to prescribe. But they were persuaded to have a medical review of their cough and a chest infection was diagnosed and treated. They left happy. Participant-25 was similarly declined benzodiazepines, but agreed to have a screening for sexually transmitted diseases.

## Discussion

### Summary

To the authors’ knowledge, this research is the first to identify and label a series of recurrent interactions between doctors and patients experiencing homelessness, which often results in exclusion of those patients from health services. The exclusion is either from them being barred or self-excluded. As patients experiencing homelessness have very poor health indices, it is important to seek to understand and reverse any process that inhibits their access to health care.

### Strengths and limitations

This research was based on qualitative input from a large number of participants from a wide range of differing homeless services. This research was conducted in Dublin and as such its findings may not be generalisable beyond the population experiencing homelessness in Dublin. The data obtained in respect of the doctor’s perspective was obtained solely from the focus group interactions. The main researcher works as a GP in specialised services for people experiencing homelessness. This may have created barriers for clients discussing issues with the researcher and also may have biased the researcher in the interpretation of the data.

### Comparison with existing literature

It has been estimated that between 15% and 30% of doctor–patient interactions can be classified as difficult encounters. Such encounters leave both doctors and patients frustrated.^38^ Exclusion from a medical service is the ultimate sanction and has been shown to be deleterious to health.^39–43^ There is extensive literature written on the importance of the doctor–patient relationship and how communication skills positively influence it.^44–46^ There is also literature that explores how the differing social-class background of healthcare providers and patients can affect the doctor–patient relationship. Providers are more likely to have negative conceptions of patients from lower socioeconomic backgrounds in terms of their personalities, abilities, behavioural tendencies, and potential for substance misuse.^47,48^ These perceptions have been demonstrated to negatively affect healthcare providers’ behaviour with such patients, including giving less information owing to the presumption that patients from lower socioeconomic backgrounds have less understanding of health issues. Patients who are perceived to be deviant are, in particular, prone to be affected by negative behaviours resulting from such healthcare provider attitudes.^14,47–49^

It is known that doctors feel ambivalent about prescribing benzodiazepines, being caught between the opposing impulses to either prescribe so as to please the patient or shorten the consultation, or not prescribe owing to the addictive potential and the desire not to been seen as an overprescriber among one’s peers.^50–52^ This ambivalence is augmented by the negative stereotypes doctors hold of drug misusers and people experiencing homelessness.^53,54^ On the patient’s side, the potential for a clash is aggravated by the fact that drug misusers feel that they, and not the doctors, are ‘experts’ on the effect of addiction on their personal lives.^55,56^ It is also recognised that mistrust does result in exclusion of patients experiencing homelessness who misuse drugs from services.^57^ Doctors have been warned to be wary of drug users‘ manipulative behaviour and to adopt a distrusting stance in such relationships. This deceit is perceived to strike at the core of the relationship between doctor and patient.^58–62^ However, if truthfulness is a requirement for doctor–patient interactions then people experiencing homelessness will always be at a disadvantage, as it is known they often have to resort to deception or ‘trickery’ in order to survive. Scamming, ruses, and criminal activity are all part of the survival strategy for people experiencing homelessness. Lee suggests that these behaviours are often effected in zones of oppression in ways where its subtlety cannot be detected. The tragedy of trickery is that people experiencing homelessness are forced through necessity to become mendacious and ‘untrustworthy’.^63^ This potential for mistrust in the doctor–patient relationship is increased by the fact that people experiencing homelessness often mistrust health services.^56,58,64–66^ The blaming conversation serves to alienate people experiencing homelessness and interferes with health service providers‘ ability to provide appropriate services.^23,59^ It is known that people experiencing homelessness have an *‘* *expectation of rejection* *,* *and anger can be quite near the surface* *...* [that] *...* *can spark off aggressive behaviour* *‘*.^56^ The assertive conversation is important as it is recognised in the literature that people experiencing homelessness are perceived as aggressive in their interactions with services.^67,68^

### Implications for research and practice

Further research could address whether such interactions are identified in other populations experiencing homelessness or drug use outside Dublin. Conversations of exclusion may be identified among other marginalised communities or in other settings, for example, education. Health professionals need to develop communication skills that will allow conversations of exclusion to change into conversations of inclusion.

This research raises the question as to whether these conversations can be transformed in practice. On the North Dublin City GP Training Programme, GP trainees are educated in an alternate approach to the benzodiazepine conversation that seeks to transform it into a conversation of inclusion. This approach is based on three presumptions as to why doctors get angry at being asked for addictive medication:

1. doctors have had negative experiences of the benzodiazepine conversation in the past;
2. doctors believe that it is unfair for a patient to ask for benzodiazepines; and
3. doctors are repeatedly told ‘bad’ doctors prescribe benzodiazepines. Thus, when patients request benzodiazepines they are asking them to be ‘bad’ doctors.

The training programme addresses these presumptions as follows. The ‘unfairness’ of the request is teased out. In this study it is pointed out how homelessness and/or drug use causes severe anxiety, panic, insomnia, and the potential to lead to severe withdrawals including seizures. The patients could argue cogently they medically need benzodiazepines. It is discussed how the system forces patients to tell lies to obtain the medical care they think they need. The fairness of the request and the fairness of refusing the request are stressed. The question becomes how to refuse without excluding.

The following approach is advised:

- First, the doctor is advised to apologise for the fact that they are not going to dispense medication and to let the patient know they understand why they feel they need the medication. (for example, *‘* *I am sorry I cannot help you as I understand why you want the tablets.’)*
- Second, the doctor ascribes the responsibility for this decision away from themselves. The doctor is usually advised to explain that they are not allowed by their Medical Council to fuel potential addictions (for example, *‘* *The Medical Council will not allow me to overprescribe these medications*.*’*)
- Third, the doctor stresses they are more than willing to help them in others ways, such as counselling, or if they have other medical complaints they can help them with. It is advised having a very positive tone and body language in stressing what they can do for the patient (for example, *‘* *But I can help you in other ways, for example ...* *‘*)
- Where the patient persists, the doctor is advised to use the broken-record technique, that is, repeat their apology and statement of understanding, and again ascribe responsibility to the Medical Council and tell the patient what they can do for them. This mantra may need to be repeated several times (for example, *‘* *I am sorry I cannot help you as ...* *‘*)

In the authors’ experience, this conversation usually results in the patient engaging with a different medical problem or leaving in a frustrated but usually non-aggressive manner. The doctor throughout this exchange is concentrating on trying to maintain the doctor–patient conversation and transform the message from ‘I cannot help you’ to ‘I really am trying to help you‘. The key point is the relationship is protected and that the patient usually feels comfortable to return for other medical issues. Thus, the conversation is transformed from one of exclusion to one of inclusion.
